# Using the Haken–Strobl–Reineker Model to Determine the Temperature Dependence of the Diffusion Coefficient

**DOI:** 10.1021/acs.jctc.4c00568

**Published:** 2024-07-17

**Authors:** William Barford

**Affiliations:** Department of Chemistry, Physical and Theoretical Chemistry Laboratory, University of Oxford, Oxford OX1 3QZ, U.K.

## Abstract

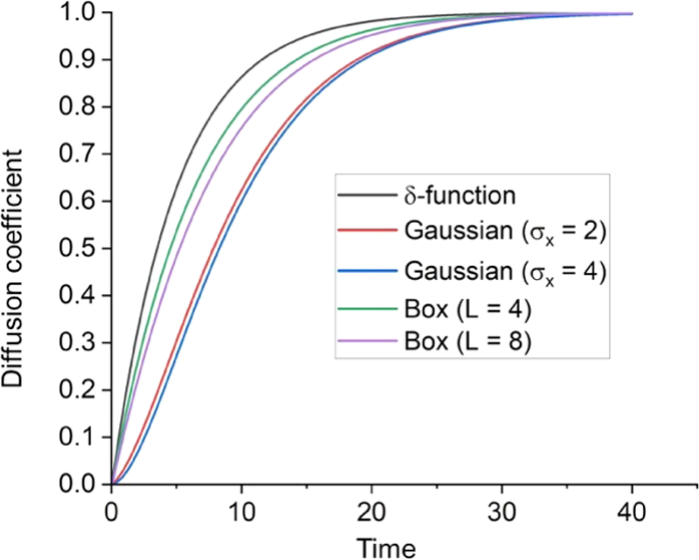

Stochastic quantum Liouville equations (SQLE) are widely used to model energy and charge dynamics in molecular systems. The Haken–Strobl–Reineker (HSR) SQLE is a particular paradigm in which the dynamical noise that destroys quantum coherences arises from a white noise (i.e., constant-frequency) spectrum. A system subject to the HSR SQLE thus evolves to its “high-temperature” limit, whereby all the eigenstates are equally populated. This result would seem to imply that the predictions of the HSR model, e.g., the temperature dependence of the diffusion coefficient, have no validity for temperatures lower than the particle bandwidth. The purpose of this paper is to show that this assumption is incorrect for translationally invariant systems. In particular, provided that the diffusion coefficient is determined via the mean-squared-displacement, considerations about detailed-balance are irrelevant. Consequently, the high-temperature prediction for the temperature dependence of the diffusion coefficient may be extrapolated to lower temperatures, provided that the bath remains classical. Thus, for diagonal dynamical disorder the long-time diffusion coefficient, *D*_∞_(*T*) = *c*_1_/*T*, while for both diagonal and off-diagonal disorder, *D*_∞_(*T*) = *c*_1_/*T* + *c*_2_*T*, where *c*_2_ ≪ *c*_1_. An appendix discusses an alternative interpretation from the HSR model of the “quantum to classical” dynamics transition, whereby the dynamics is described as stochastically punctuated coherent motion.

## 1 Introduction

Coherent exciton dynamics in static, ordered molecular systems was described by Merrifield^1^ in 1958. Assuming an exciton created at time *t* = 0 on a monomer, say *n* = 0, he showed that the subsequent wave function is Ψ_*n*_(*t*) = *J*_*n*_(2β*t*), where *J*_*n*_ is the *n*th order Bessel function of the first-kind and β is the intermonomer exciton transfer integral. The wave function (illustrated in Appendix A) spreads ballistically with a constant speed and a mean-squared-displacement (MSD) increasing quadratically with time.

As Merrifield observed,^1^ however, dynamics on a static, ordered system is an idealization. Various physical processes, e.g., exciton–phonon coupling, and static and dynamic disorder destroy the coherent motion, eventually causing incoherent (or diffusive) motion where the MSD increases linearly with time. This topic now has a long and rich history, with many reviews describing the state of the field.^2−6^

The purpose of this paper is to expand on one particular rich area of investigation, namely the role of thermally induced noise in destroying quantum coherences. A notable paradigm in this subject is the so-called HSR stochastic quantum Liouville equation (SQLE), developed and investigated by Haken, Strobl and Reineker.^2,7^ This equation was developed from the underlying time-dependent Schrödinger equation (TDSE) assuming that the dynamical fluctuations obey a white-noise spectrum, i.e., a constant power spectrum (or an Ohmic spectral function). Many important results have been derived from this model.^2,8,9^ In particular, it describes the “quantum to classical” transition, in which exciton dynamics exhibits a crossover from ballistic to diffusive behavior as a result of the noise destroying the coherent motion.

As stated, the HSR model assumes a white-noise spectrum, which implies that quantum transitions can occur between any pair of system energy eigenstates. This is turn implies that a system subject to the HSR SQLE evolves to its “high-temperature” limit, whereby all the eigenstates are equally populated. This result would seem to suggest that the predictions of the HSR model, e.g., the temperature dependence of the diffusion coefficient, have no validity for temperatures lower than the particle bandwidth. The purpose of this paper is to show that this assumption is incorrect for translationally invariant systems. In particular, provided that the diffusion coefficient is determined via the mean-squared-displacement, considerations about detailed-balance are irrelevant. Consequently, the high-temperature prediction for the temperature dependence of the diffusion coefficient may be extrapolated to lower temperatures, provided that the bath remains classical.

This key result will be proved in Section 2. Since the HSR SQLE predictions for the diffusion coefficient in translationally invariant systems are valid for temperatures lower than the particle bandwidth, the predictions of the underlying stochastic TDSE are also equally valid. We use this realization to reinterpret the quantum to classical transition as stochastically punctuated coherent motion. This is described in Appendix A.

Unlike the use of the MSD, Appendix B shows that the velocity autocorrelation function cannot be used to extrapolate the HSR predictions to temperatures lower than the bandwidth. Finally, Appendix C derives an expression for the temperature-dependence of the dephasing rate and Appendix D contains some details of the computational techniques.

## 2 Theory

### 2.1 Model of Exciton Dynamics in Linear Molecular Systems

We formulate the problem in terms of Frenkel exciton dynamics in one-dimensional molecular systems, e.g., J-aggregates or conjugated polymers. However, the analysis applies equally to triplet excitons and charges.

The total Hamiltonian is
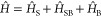
1where *Ĥ*_S_, *Ĥ*_SB_ and *Ĥ*_B_ are the system, system-bath and bath Hamiltonians, respectively. *Ĥ*_B_ is defined in Appendix C (eq 47).

The system Hamiltonian is defined as
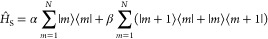
2The ket |*m*⟩ represents an exciton on monomer *m*, denoting a “site”, where *N* is the number of sites. α and β are the onsite potential and nearest-neighbor exciton transfer integral, respectively.

For a system with translational invariance, the eigenstates of *Ĥ*_S_ are the Bloch states
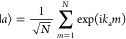
3with eigenvalues *E*_a_ = α + 2β cos *k*_a_, where *k*_a_ = 2π*a*/*N* is the wavevector. The quantum numbers that label the eigenstates satisfy 1 ≤ *a* ≤ *N*. The particle bandwidth in one-dimension is 4|β|.

*Ĥ*_SB_ is the system-bath Hamiltonian
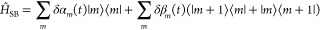
4where δ*α*_*m*_(*t*) and δ*β*_*m*_(*t*) represent dynamical fluctuations in α and β. These fluctuations are assumed to be uncorrelated in space, i.e.,
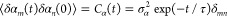
5and
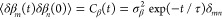
6In addition, in the white noise limit, defined by σ_×_τ ≪ 1, the bath correlation functions *C*_×_(*t*) → 2γ_×_ℏ^2^δ(*t*), where γ_×_ = σ_×_^2^τ/ℏ^2^ and × indicates α or β. White noise means that the power spectrum, *I*(ω), is constant (or the spectral function, *J*(ω) ∼ ω·*I*(ω) ∼ ω, i.e., Ohmic), which implies that the time-dependent part of the Hamiltonian induces transitions between all pairs of eigenstates.

For a classical, harmonic bath, γ_×_ ∝ σ_×_^2^ ∝ *k*_B_*T*. More specifically, as shown in Appendix C, for linear system-bath coupling
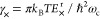
7where *E*_×_^r^ is the reorganization energy arising from the system-bath coupling and ω_c_ is a high-frequency cutoff for the spectral function.

### 2.2 Determining the Temperature-Dependent Diffusion Coefficient

The one-dimensional thermal diffusion coefficient as a function of time is defined as
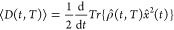
8where ⟨···⟩ indicates a thermal average, *x̂* is the operator for the particle position, and ρ̂(*t*, *T*) is the system’s reduced density operator. In the long-time limit the asymptotic diffusion coefficient is
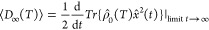
9where
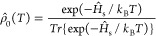
10is the equilibrium density operator.

Evaluating the trace over the eigenstate basis of *Ĥ*_S_, eq 9 becomes
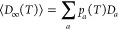
11where
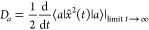
12and *p*_*a*_(*T*) is the Boltzmann factor.

The mean-squared-displacement of a particle prepared in an arbitrary state |ψ⟩ at *t* = 0 is defined as
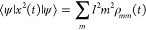
13where  is the intermonomer separation and ρ_*mm*_(*t*) are the diagonal elements of the system density matrix in the site basis, {|*m*⟩}. As described in Section 2.3, the density matrix, ρ_*mn*_(*t*), evolves according to an appropriate quantum Liouville equation with the initial condition ρ_*mn*_(0) = ⟨*m*|ψ(0)⟩⟨ψ(0)|*n*⟩.

Transforming to the eigenstate basis via a transformation matrix, **S**, eq 13 becomes
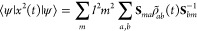
14where ρ̃_*ab*_ is the density matrix in the eigenstate basis and *S*_*ma*_ = ⟨*m*|*a*⟩. For a system with translational invariance, the transformation matrix elements are the Bloch factors
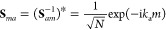
15

Splitting the double sum in eq 14 over *a* and *b* into the separate sums of *a* = *b* and *a* ≠ *b*, and using eq 15 we obtain
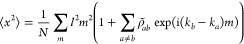
16where we have also used ∑_*a*_ ρ̃_*aa*_ = 1. The significance of this result is that it shows that for a translationally invariant system the diffusion coefficient of a particle prepared in the state |ψ⟩ depends on the evolution of eigenstate coherences and not directly on the evolution of eigenstate populations. Moreover, as will be shown in Section 2.3, for a translationally invariant system, eigenstate coherences are decoupled from eigenstate populations, and thus for such systems the diffusion coefficient is completely independent of eigenstate populations. This means that when evaluating eq 11 to determine ⟨*D*_∞_(*T*)⟩ enforcing detailed balance—or ensuring that eigenstate populations satisfy their thermal values—is unnecessary. Thus, ⟨*D*_∞_(*T*)⟩ only depends on temperature parametrically via the temperature-dependence of the dephasing rate, eq 7.

Finally, as shown in Section 2.3, the asymptotic diffusion coefficient is independent of the initial state. Denoting this asymptotic value by *D*_∞_(*T*), setting *D*_*a*_ = *D*_∞_(*T*), ∀*a*, and using ∑_*a*_*p*_*a*_ = 1, eq 11 becomes ⟨*D*_∞_(*T*)⟩ = *D*_∞_(*T*). Thus, our task now is to determine *D*_∞_(*T*) for a given dephasing rate and for arbitrary initial conditions. This is achieved via the Haken–Strobl–Reineker model as described in the next section.

### 2.3 The Haken–Strobl–Reineker Model

Haken and Strobl showed that ensemble averages of observables determined via the stochastic TDSE may be evaluated by a SQLE.^2,7^ For the case of diagonal noise within the site basis, the SQLE reads
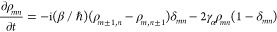
17Rotating to the eigenstate basis of *Ĥ*_S_ via eq 15, the SQLE becomes for the populations
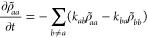
18and for the coherences
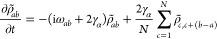
19where ω_*ab*_ = (*E*_*a*_ – *E*_*b*_)/ℏ.

From eqs 18 and 19 we note the following:

1. In the HSR model *k*_*ab*_ = *k*_*ba*_ = 2γ_α_/*N*, thus guaranteeing equal eigenstate populations in the long-time limit. In principle, one could impose detailed balance on the rates, thus ensuring that the populations equilibrate to their thermal values. However, as now shown, this is unnecessary.
2. The equations of motion for the populations and coherences are decoupled. This proves, by virtue of eq 16, that ⟨*D*_∞_(*T*)⟩ is independent of temperature-dependent populations and only depends on *T* parametrically via γ_α_(*T*).
3. Equation 18 is a single *N*-coupled equation, whereas eq 19 are (*N* – 1) × *N*-coupled equations. A numerical solution is described in Appendix D.

## 3 Results

Haken, Reineker and co-workers derived an expression for *D*(*t*) for an initial δ-function source.^2,10^ For diagonal noise this is
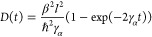
20Reineker^2^ also derived the asymptotic value, *D*_∞_, for arbitrary initial conditions. For diagonal noise
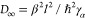
21

**Figure 1:**
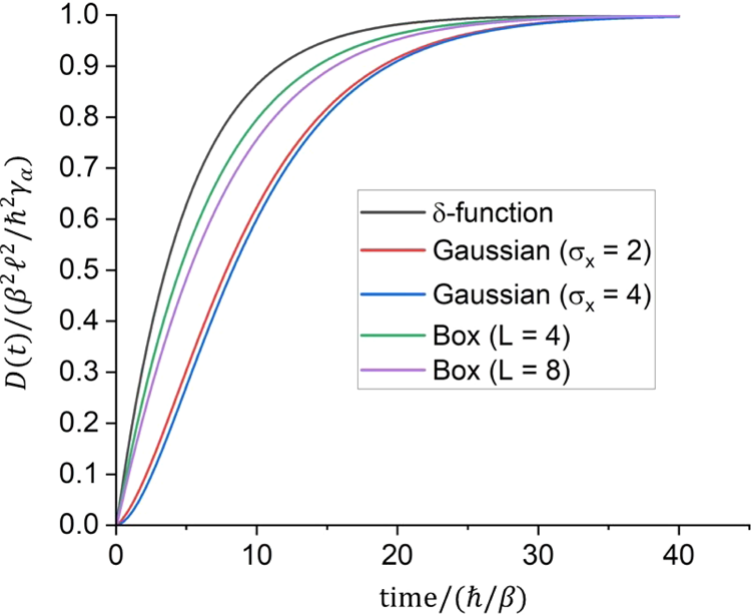
Computed *D*(*t*) using eq 19 for various initial conditions and diagonal white noise, γ_α_ = 0.1β/ℏ. For the δ-function source, *D*(*t*) satisfies eq 20. The asymptotic values satisfy satisfies eq 21. The Gaussian wave functions are defined by eq 27. The particle-in-a-box wave functions are ψ_*n*_ = (2/(*L* + 1))^1/2^ sin(π*n*/(*L* + 1)). The diffusion coefficient at short times for the Gaussian wave functions passes through a superlinear regime, as also reported in ref (11).

These analytical predictions were confirmed by solving both the HSR SQLE and the stochastic TDSE numerically (as described in Appendix D). Figure 1 shows the numerically evaluated *D*(*t*) for different initial conditions, namely a δ-function, Gaussian and particle-in-a-box sources. The δ-function result satisfies eq 20, while for all other sources, having a larger initial mean-squared-size and thus a smaller initial mean-squared-speed, the diffusion coefficient increases more slowly with time. However, eq 21 is satisfied for all initial conditions.

For translationally invariant systems, the evolution of the populations and coherences are also decoupled in the presence of off-diagonal noise. Thus, for both diagonal and off-diagonal noise, the high-temperature limit determined from the HSR SQLE can be extrapolated to temperatures lower than the bandwidth. Reineker^2^ showed that for arbitrary initial conditions^12^
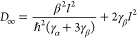
22where γ_α_ and γ_β_ are the diagonal and off-diagonal dephasing rates, respectively.

Substituting the temperature-dependence of the dephasing rates given by eq 7, we thus have the following prediction for the temperature-dependence of the diffusion coefficient in the presence of white noise:
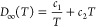
23where
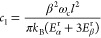
24and
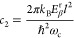
25

Equation 23 is valid for translationally invariant systems subject to white noise for all temperatures—including temperatures lower than the particle bandwidth, 4|β|—provided that the bath remains classical. It remains valid if uniform long-range couplings are included, although the coefficients *c*_1_ and *c*_2_ are altered.

## 4 Conclusions

This paper has shown that for translationally invariant systems the predictions of the HSR model for the thermal diffusion coefficient can be extrapolated to temperatures lower than the particle bandwidth, provided that the bath remains classical. The proof relies on the observation that for such systems the mean-squared-displacement is independent of eigenstate populations. Consequently, considerations about detailed balance are irrelevant, and thus the diffusion coefficient depends on temperature only parametrically via the dephasing rates. When diagonal disorder dominates, *D*(*T*) ∼ *T*^–1^. This high-temperature limit for *D*(*T*) is a common prediction in many theories of charge and energy transport.^13^

Translationally invariant systems subject to white noise are an idealization of more realistic systems, where static disorder, correlated (non-Markovian) noise and electron–phonon interactions causing polaron formation are all important processes that will modify the predictions of this paper. For such systems, numerically solutions of the TDSE or QLE should explicitly ensure that detailed balance and stationarity are maintained during the system’s evolution (e.g., the Redfield quantum Liouville equation of motion,^4,14−16^ the time-dependent wavepacket diffusion method,^17,18^ hierarchical equations for open systems,^19^ and stochastic Liouville equation methods^20^). The recently proposed MASH surface-hopping schemes^21,22^ are also promising techniques for such simulations.

We conclude by noting that in the presence of static, diagonal disorder the scaling of *D*(*T*) with temperature deviates from the HSR prediction.^8,9,23^ In particular, for γ ∼ *T* < β, *D*(*T*) ∝ *T*. This increase of the diffusion coefficient with temperature at low temperatures, sometimes known as environment-assisted quantum transport,^8^ is a consequence of the thermal fluctuations destroying the Anderson localization arising from coherent superposition of the particle wave function.

## Figures and Tables

**Figure 2:**
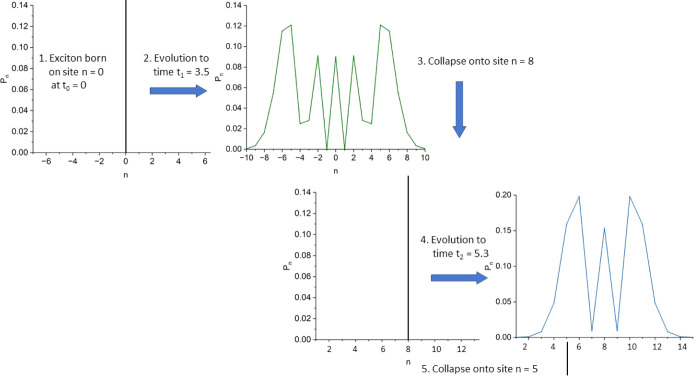
An illustration of the evolution of a particle wave function subject to the TDSE with stochastic wave function collapses caused by white noise. The particle probability, *P*_*n*_(*t*) = *J*_*n*_^2^(2β*t*). The cumulative effect of the stochastic wave function collapses is to cause particle diffusion, where *D* = β^2^/γ_α_, in exact agreement with the HSR model. γ_α_ = 0.1β/ℏ. Time is in units of ℏ/β.

**Figure 3:**
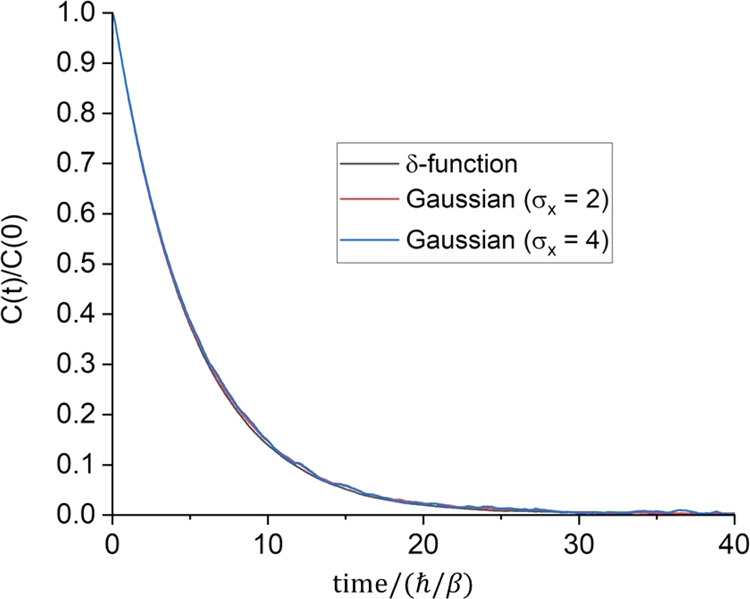
Velocity autocorrelation function, *C*(*t*) = ⟨ψ|*Ĉ*_*v*_(*t*)|ψ⟩, where *Ĉ*_*v*_(*t*) = *v̂*(*t*)*v̂*(0), computed via the stochastic TDSE with diagonal white noise (γ_α_ = 0.1β/ℏ). *C*(*t*) satisfies eq 43 for any initial condition. *C*(0) = ⟨*v̂*⟩_0_ is  for a δ-function, while it is β^2^/ℏ^2^σ_*x*_^2^ for a Gaussian wavepacket (defined in eq 27), with σ_*x*_ in units of  and *v̂* is defined in eq 60.

## Appendix A The Quantum to Classical Transition

This appendix discusses an alternative interpretation from the HSR model of the “quantum to classical” dynamics transition, whereby the dynamics is described as stochastically punctuated coherent motion.

In free space the one-dimensional TDSE reads

26

where *m* is the particle mass. As is well-known,^15,24^ a particle prepared in a Gaussian wavepacket at *t* = 0, i.e.,

27

undergoes a ballistic (or coherent) spread. Thus, its MSD, σ_*x*_^2^(*t*), increases as

28

In contrast, the diffusion equation describes the evolution of the density of an ensemble of particles, ρ(*x*, *t*). In free space the one-dimensional diffusion equation reads

29

Although this equation has precisely the same mathematical form as eq 26, the absence of imaginary i means that its physical predictions are quite different. In particular, an initially localized distribution of particles spreads diffusively, i.e.,

30

where the MSD increases as

31

The HSR equation describes how white noise causes a quantum particle’s coherent motion to become incoherent. As shown by Reineker,^2^ assuming a δ-function source on a lattice, i.e., |Ψ_*n*_(0)|^2^ = δ_*n*0_, for *t* ≪ 2γ,

32

(as predicted by the TDSE on a lattice^1^) where *J*_*n*_ is the *n*th-order Bessel function of the first kind. In contrast, for *t* ≫ 2γ,

33

(as predicted by the diffusion equation) where *D* = σ_*x*_^2^(*t*)/2*t* = β^2^/γ_α_ for diagonal noise. In general for a δ-function source (where henceforth in this Appendix, we set )^2,25^

34

In this interpretation, noise causes a crossover from coherent to incoherent motion. A different interpretation of the quantum to classical transition, however, is afforded by the Lindblad formulation of the quantum Liouville equation. As we now show, in this interpretation the particle’s dynamics can be viewed as stochastically punctuated coherent motion.

1. The Lindblad dissipator is^16^
  35
  where *Â*_*m*_ is the Lindblad jump operator. For diagonal white-noise,^8^*Â*_*m*_ = |*m*⟩ ⟨*m*| and Γ = 2γ_α_.
2. An ensemble of quantum trajectories reproduces the observables obtained via a QLE if each trajectory undergoes nonunitary evolution via an effective nonhermitian Hamiltonian,^26^ defined by
  36
3. The simulation of a quantum trajectory then proceeds as follows:
  (a) Given |Ψ(*t*)⟩ at a time *t*, compute
    37
    where δ*t* is the time step. (See Appendix D for details.)
  (b) Determine δ*p*, defined via ⟨Ψ_trial_|Ψ_trial_⟩ = 1 – δ*p*.
  (c) Then,
    (i) with a probability (1 – δ*p*) define
      38
      In this case the wave function evolves according to *Ĥ*_eff_.
      Or,
    (ii) with a probability δ*p* define
      39
      Using the definition that *Â*_*m*_ = |*m*⟩ ⟨*m*|, eq 39 implies that |Ψ(*t* + δ*t*)⟩ = |*m*⟩. Thus, in this case, the wave function collapses onto site *m*.
  (d) If a quantum jump occurs in 3(c)(ii), the site *m* is chosen with a probability *P*_*m*_ = |Ψ_*m*_(*t*)|^2^.

Thus, the effect of the Lindblad jump operator in step 3(c)(ii) is to collapse the coherently evolving wave function onto the site *m*, after which it then again expands coherently until the next collapse. This process is illustated in Figure 2: At time *t* = 0 the particle is created on site *n*_0_ = 0. Its wave function then evolves coherently until the first quantum jump at *t* = *t*_1_. For *t* ≤ *t*_1_, Ψ_*n*_(*t*) = *J*_*n*_(2β*t*), where *J*_*n*_ is the *n*th order Bessel function.^1^ At *t* = *t*_1_ the wave function collapses onto site *n* = *n*_1_, whereupon it again evolves coherently until a time *t*_2_, when it again collapses onto site *n* = *n*_2_.

As these collapses are stochastic in space and time, their cumulative effect is to cause particle diffusion. We can determine the diffusion coefficient as follows. For a diffusive process the MSD is defined as

40

where *N*(*t*) = *t*/⟨τ⟩ is the number of jumps in a time *t* and ⟨τ⟩ = 1/Γ = 1/2γ_α_ is the average time interval between jumps.  is the mean-squared jump size, determined by , where *v* is the particle’s coherent speed. For a δ-function source on a lattice, *v* = √2β. As determined by simulating the TDSE with Lindblad jump operators, for the dynamics described here, ⟨τ^2^⟩ = 2⟨τ⟩^2^ = 2/(2γ_α_)^2^. Hence,  and therefore , thus—on average—rederiving the prediction of the HSR quantum Liouville equation. Ensemble averaging over many particle trajectories will give precisely the same observables (e.g., average particle density) as the stochastic quantum Liouville approach, but the interpretation of the dynamics is different, namely stochastically punctuated coherent dynamics.

## Appendix B Using the Velocity Autocorrelation Function

An alternative definition to the thermal diffusion coefficient from eq 8 is

41

where *Ĉ*_*v*_(*t*) = *v̂*(*t*)*v̂*(0) is the velocity autocorrelation operator. The derivation of eq 41 from the identity

42

assumes stationarity, i.e., that the time-average of *v*(*t* + *t*′)*v*(*t*′) is independent of *t*′.

However, if ⟨ψ|*Ĉ*_*v*_(*t*)|ψ⟩ is evaluated for an arbitrary state |ψ⟩ via the HSR SQLE the stationarity condition is not met. This is because, unlike the case for the mean-squared-displacement, ⟨ψ|*Ĉ*_*v*_(*t*)|ψ⟩ depends on eigenstate populations, which become equally populated under evolution via the HSR SQLE.

Indeed, as shown in Figure 3,

43

in agreement with the definition^9,14^

44

for the HSR model. Integrating eq 43 over time gives

45

which erroneously predicts that the asymptotic diffusion coefficient depends on the initial condition.

Equation 45 is only correct for a δ-function source, because this maximally localized in real-space wave function is maximally delocalized in momentum-space, i.e., all the energy (momentum) eigenstates of a translationally invariant system are already equally populated at *t* = 0 so that the stationarity condition is automatically satisfied. Otherwise, for arbitrary initial conditions, eq 41 is only valid provided detailed balance is enforced. Such a scenario is described in ref (14).

## Appendix C The Dephasing Rate

This appendix describes a derivation of eq 7.

For linear system-bath coupling, the system-bath Hamiltonian is^15^

46

while the bath Hamiltonian of harmonic oscillators is^15^

47

*u*_*j*_ are the mass-weighted oscillator displacements and *c*_*j*_^×^ are the weightings of each normal mode with associated spectral functions,

48

The bath autocorrelation function associated with diagonal noise is *C*_α_(*t*) = ⟨δ*α*(*t*)δ*α*(0)⟩, where δ*α*(*t*) = ∑_*j*_*c*_*j*_^α^*u*_*j*_(*t*) is the dynamical fluctuation of the on-site potential energy. For a classical, harmonic bath and linear system-bath coupling^15^

49

The spectral function, *J*_α_(ω), for an Ohmic bath with a high-frequency cutoff, ω_c_ is defined as

50

*E*_α_^r^ is the reorganization energy of the bath modes, satisfying

51

Thus, using

52

as ω_c_ → ∞, we obtain

53

Equating eq 53 with the definition of γ_α_ given in Section 2.1, i.e., *C*_α_(*t*) = 2γ_α_ℏ^2^δ(*t*), yields an expression for the dephasing rate which is linearly proportional to temperature:

54

## Appendix D Computational Methods

### D.1 Numerical Solution of the HSR Stochastic Quantum Liouville Equation

Since the equation of motion for the coherences in the eigenstate basis, eq 19, are block diagonal with respect to the quantum number *c*, there are (*N* – 1) × *N*-coupled equations. These can be cast into the general form

55

for 1 ≤ *i* ≤ (*N* – 1), where **L** is the coupling matrix. The solution from linear algebra is

56

where **S** is the matrix whose columns are the eigenvectors of **L**, {λ} are the corresponding eigenvalues and *P*_*k*_(0) is an initial condition. To produce the results illustrated in Figure 1, the complex matrix **L** was diagonalized via the LAPACK routine ZGEEV, and the complex matrix **S** was inverted via the LAPACK routines ZGETRF and ZGETRI.

### D.2 Numerical Solution of the Time-Dependent Schrodinger Equation

In general, given Ψ(*t*) we require

57

This is easily accomplished via the short iterative Lanczos propagator method,^24^ whereby a Krylov space of *N* vectors is generated via the Lanczos method. Diagonalizing the tridiagonal Hamiltonian, **H**_Lanczos_, within the Krylov space yields

58

where **S** is the is the matrix whose columns are the eigenvectors of **H**_Lanczos_, the diagonal elements of **D** are its eigenvalues, and Ψ(*t*) is the first vector in the Krylov space.

The expectation value of the velocity autocorrelation function is

59

where |Φ⟩ = *v̂*|Ψ⟩ and the velocity operator on a lattice is

60
